# Low-cost, ultracompact handheld optical coherence tomography probe for *in vivo* oral maxillofacial tissue imaging

**DOI:** 10.1117/1.JBO.25.4.046003

**Published:** 2020-04-20

**Authors:** Kaiyan Li, Zihan Yang, Wenxuan Liang, Jianwei Shang, Yanmei Liang, Suiren Wan

**Affiliations:** a Southeast University, School of Biological Science and Medical Engineering, Nanjing, Jiangsu, China; b Nankai University, Institute of Modern Optics, Tianjin, China; c Columbia University, Mortimer B. Zuckerman Mind Brain Behavior Institute, New York, United States; d Nankai University, Tianjin Stomatological Hospital, Hospital of Stomatology, Department of Oral Pathology, Tianjin, China

**Keywords:** optical coherence tomography, handheld probe, low cost, ultracompact, oral maxillofacial tissue

## Abstract

**Significance:** Optical coherence tomography (OCT) has proven useful for detecting various oral maxillofacial abnormalities. To apply it to clinical applications including biopsy guidance and routine screening, a handheld imaging probe is indispensable. OCT probes reported for oral maxillofacial imaging were either based on a bulky galvanometric mirror pair (not compact or long enough) or a distal-end microelectromechanical systems (MEMS) scanner (raised safety concerns), or adapted from fiber-optic catheters (ill-suited for oral cavity geometry).

**Aim:** To develop a handheld probe featuring great compactness and excellent maneuverability for oral maxillofacial tissue imaging.

**Approach:** A dual-axis MEMS scanner was deployed at the proximal end of the probe and the scanned beam was relayed to the distal end through a 4f configuration. Such design provides both a perfect dual-axis telecentric scan and excellent compactness.

**Results:** A handheld probe with a rigid part 70 mm in length and 7 mm in diameter and weighing 25 g in total was demonstrated through both *ex vivo* and *in vivo* experiments, including structural visualization of various oral maxillofacial tissues and monitoring the recovery process of an oral mucosa canker sore.

**Conclusions:** The proposed probe exhibits excellent maneuverability and imaging performance showing great potential in clinical applications.

## 1 Introduction

Oral maxillofacial conditions are highly prevalent and have become significant public health challenges worldwide.^1^^,^^2^ Given the vital role played by oral maxillofacial regions both functionally and esthetically, false diagnosis and subsequently delayed intervention or mistreatment can impose on patients not only heavy economic burden but also great social psychological stress due to potential malnutrition and disfiguring.^3^^,^^4^ Reliable diagnosis is crucial to formulating tailored treatments and early intervention to effectively prevent further deterioration and to greatly improve the overall outcome and life qualities of patients.^1^^,^^5^ Histopathological interrogation of biopsy specimens is still the current gold standard of definitive diagnosis of oral maxillofacial lesions. However, the biopsy procedure, typically guided by an upper endoscope, is prone to sampling errors^6^ since in many cases, abnormal tissue structures emerge from subepithelial locations [e.g., basement membrane (BM) for basal cell carcinoma] in premalignant stages,^7^^,^^8^ and exhibit barely discernible visual difference on the tissue surface. Standard medical imaging modalities, including x-ray, computed tomography (CT), and ultrasonography, are not well-suited for real-time biopsy guidance due to their high cost, limited imaging resolution, and insufficient detection sensitivity of early transformation.^9^ Therefore, there has been compelling need for a noninvasive, high-resolution, and cost-effective imaging technology that is able to visualize inconspicuous subepithelial microscopic abnormalities over an extended area *in vivo* and in real time and can be used for routine screening or targeted biopsy guidance.

Optical coherence tomography (OCT), featuring intrinsic depth-resolved capability, high resolution, considerable tissue penetration depth, and rapid volumetric imaging speed, has become an exceptional three-dimensional (3-D) microscopy technology for label-free, noninvasive imaging of biological tissues *in vivo*.^10^^,^^11^ Powered by a broadband near-infrared light source, OCT can visualize tissue microstructures up to 2 mm below the tissue surface with an axial resolution of ∼10  μm. The high-volumetric speed further enables OCT to interrogate relatively large areas in a short period with minimal motion artifacts. The feasibility of using OCT for differentiating abnormal oral maxillofacial tissues and for post-treatment follow-up examinations has been evaluated and confirmed repetitively.^12^^–^^15^ For *in vivo* scanning, a handheld probe is required. In essence, the shape irregularity of the oral cavity demands that the probe should be (1) compact enough to access all sites within the oral cavity and (2) sufficiently light for easy manipulation and stability; routine screening additionally demands that an ideal OCT probe should better be (3) cost-effective and (4) fast enough to minimize motion artifacts. The beam scanner is the central component and limiting factor of the compactness of an OCT probe. Three types of scanning mechanisms have been explored in oral cavity OCT probes hitherto reported: (1) single- or dual-galvanometer mirror(s), (2) microelectromechanical systems (MEMS) scanner, and (3) fiber-optic rotary pullback scanning. As summarized in Table 1, galvanometer scanners, comprising two orthogonally oriented X and Y mirrors mounted on servo motors, are generally bulky and costly.^16^^–^^19^ Fiber-optic rotary pullback scanning catheter, despite its excellent compactness, is designed for side-viewing imaging of cylindrical lumens and therefore, when applied to oral maxillofacial imaging, yields meaningful data over around half of the scanning circumference,^23^ leading to significant waste of both acquisition bandwidth and computational resources. In addition, the fiber-tethering nonrigid design does not lend itself to easy manipulation. MEMS scanner-based probes were also developed as alternatives to reduce probe size and cost. Almost all previous prototypes deploy a two-axis MEMS mirror at the distal end to deflect the beam in a side-viewing geometry,^24^^–^^26^^,^^29^^,^^30^ which complicates the distal-end design and implementation, and brings safety concerns as high-voltage drive signals are delivered close to the tissue under interrogation.

**Table 1:** Technical summary of published OCT probes designed for *in vivo* oral imaging.

Reference	Scanning mechanism	Scanner location	Rigid part dimension (mm)	FOV (mm)	Viewing	Resolution (μm) lateral × axial
Davoudi et al.^16^	XY galvo	Proximal	170 (long) × 20 (diameter)	2×2	Forward	19×7 (in air)
Choi and Wang^17^	XY galvo	Proximal	27 (long) × 10 (diameter)	2×2	Side	22×20.9 (in air)
			23 (long) × 5 (diameter)	2×2	Forward	22×20.9 (in air)
Wang et al.^18^	XY galvo	Proximal	120 (long) × 13 × 8	4.6×4.6	Side	12×12 (in air)
Tsai et al.^19^	XY galvo	Proximal	92 (long) × 10 (diameter)	2×2	Forward	10×8
Walther et al.^20^	XY galvo	Proximal	200 (long) × 10 (diameter)	4.8×4.8	Forward	17.5×11.6
Yoon et al.^21^	XY galvo	Proximal	180 (long) × 10 (diameter)	5.5 (diameter)	Forward	15×12.5 (in air)
Higgins and Pierce^22^	X galvo	Proximal	126 (long) × 19 (tapered tip >5) (diameter)^a^	2^b^ (B-frame)	Forward	8.0×9.3
Lee et al. ^23^	Rotary-pullback	Proximal (w/ FORJ)	1.5 outside diameter catheter	2.34 (circ) × 90 (pull)	Side	20 to 40 lateral
Aguirre et al.^24^	Two-axis MEMS	Distal	25 (long) × 5 (diameter)	1.8×1.0	Side	12×4 (in tissue)
Wang et al.^25^	Two-axis MEMS	Distal	15 (long) × 3.5 (diameter)	2×2	Side	17.5×10.6 (in air)
Sun et al.^26^	Two-axis MEMS	Distal	12 (long) × 5.8 (diameter)	2.3×2.3	Side	20×10 (in air)
Kim et al.^27^	Two-axis MEMS	Distal	12 (long) × 2.8 (diameter)	1.5×1	Side	23×11.27 lateral
Lenton et al.^28^	Two-axis MEMS	Proximal	78 (long) × 16^c^	5.2×3.5^b^	Side	80×11 (air)
Li et al. (this work)	Two-axis MEMS	Proximal	70 (long) × 7 (diameter)	2×2	Forward	10×17 (air)

Note: FORJ, fiber-optic rotary joint.

a Diameter inferred from figures with given scale bar.

b FOV inferred from figures within respective articles.

c Rigid length inferred from figures within the article.

Here, we present an alternative design of compact OCT probe for oral maxillofacial imaging, by deploying a two-axis MEMS scanner at the proximal end of the probe and relaying the scanned beam to the distal end through a 4f configuration. Compared with XY galvanometric mirror pair, this MEMS-based scanner is more compact and lower cost. Compared with designs that deploy the MEMS scanner at the distal end, such proximal end deployment significantly relaxes the compactness requirement on a MEMS device, allowing the usage of a larger MEMS device with improved optical and mechanical performance. In addition, deploying the MEMS at the proximal end avoids delivering high-voltage signals to the distal end and reduces the risk of electric shock. More importantly, our design conjugates both scan axes to the back focal plane of the objective, ensuring a true telecentric scan and faithful volumetric reconstruction. Such excellent telecentricity is unavailable on concatenated orthogonal galvanometer mirror-based or distal-end MEMS scanner-based designs. Following these principles, we prototyped an ultracompact pencil-like (7 mm in diameter), low-cost, forward-viewing handheld OCT probe with a total weight of only 25 g and very fast volumetric imaging speed for oral maxillofacial screening. The outstanding performance and versatility of the probe for multipurpose imaging were demonstrated through real-time and *in vivo* imaging of various human oral cavity tissues.

## 2 Design and Characterization

### 2.1 Low-Cost, Ultracompact Handheld Probe Design and Assembly

**Fig. 1:**
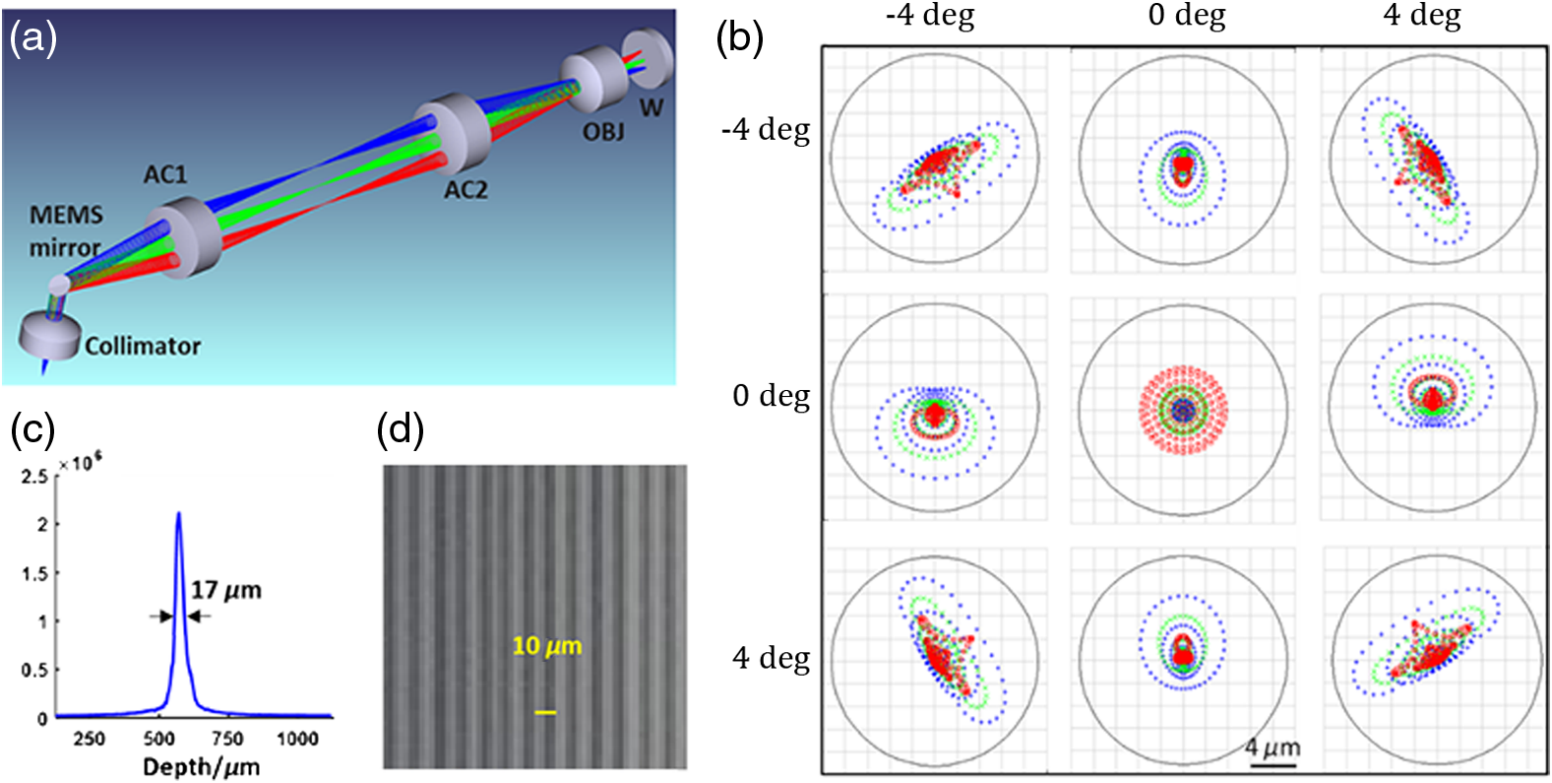
Optical design and characterizations of the ultracompact handheld OCT probe. (a) Optical schematic of the probe consisting of a collimator, a MEMS mirror, two achromatic relay lenses (AC1 and AC2), an achromatic objective (OBJ), and an antireflection window (W). Beams scanning at different angles are coded by different colors. (b) Spot diagrams (in air) of the focused beam spanning an FOV of ±4  deg. Black circles surrounding beam spots (color coded according to wavelength, blue: 1260 nm, green: 1310 nm, and red: 1360 nm) represent the corresponding Airy disks. (c) Measured axial PSF profile. (d) *En face* image of a resolution test target.

Figure 1(a) shows the schematic optical design of the ultracompact handheld probe. Light from a single-mode fiber is collimated and reflected by a 2-mm-diameter MEMS mirror (A7M20.1, Mirrorcle Tech. Inc.) into a 4f relay telescope composed of two identical achromats (AC064-015-C, Thorlabs). The utilization of a two-axis MEMS scanner is the key of the entire probe. For a traditional galvo-scanner pair, the X scan mirror needs to be conjugated to the Y scan mirror to implement a perfect telecentric scan. Such conjugation requires a well-designed relay telescope, which is not conducive to miniaturization. The MEMS scanner features a single scan mirror that can deflect along both x and y directions with a common pivot point, therefore allowing doubly telecentric scanning with minimal interaxis cross talk or spatial resolution variation throughout the field of view (FOV). The focal length of the aspheric collimator (F230APC-1310, Thorlabs, f=4.59  mm) is chosen so that the collimated beam (1.2 mm in $1/e^{2}$ diameter) is not clipped by the MEMS mirror viewed from 45 deg. All lenses including the achromatic objective (AC050-008-C, Thorlabs) are commercially available. According to Zemax simulation, ±4  deg two-dimensional scanning angle of the MEMS scanner can cover an FOV of $2\times 2\;{\mathrm{mm}}^{2}$ (as used in all experiments below), with a near-diffraction limited resolution of ∼9  μm maintained throughout the entire FOV [Fig. 1(b)]. By steadily translating the probe at a constant speed, a larger extended volumetric FOV can be obtained in a panoramic fashion. A representative measured axial point spread function (PSF) is shown in Fig. 1(c), revealing an axial FWHM resolution of ∼17  μm (in air). The lateral resolution was evaluated from an *en face* image of a grating-like resolution target. As shown in Fig. 1(d), line pairs of 10  μm in period are clearly resolved, confirming that the lateral resolution is better than 10  μm.

**Fig. 2:**
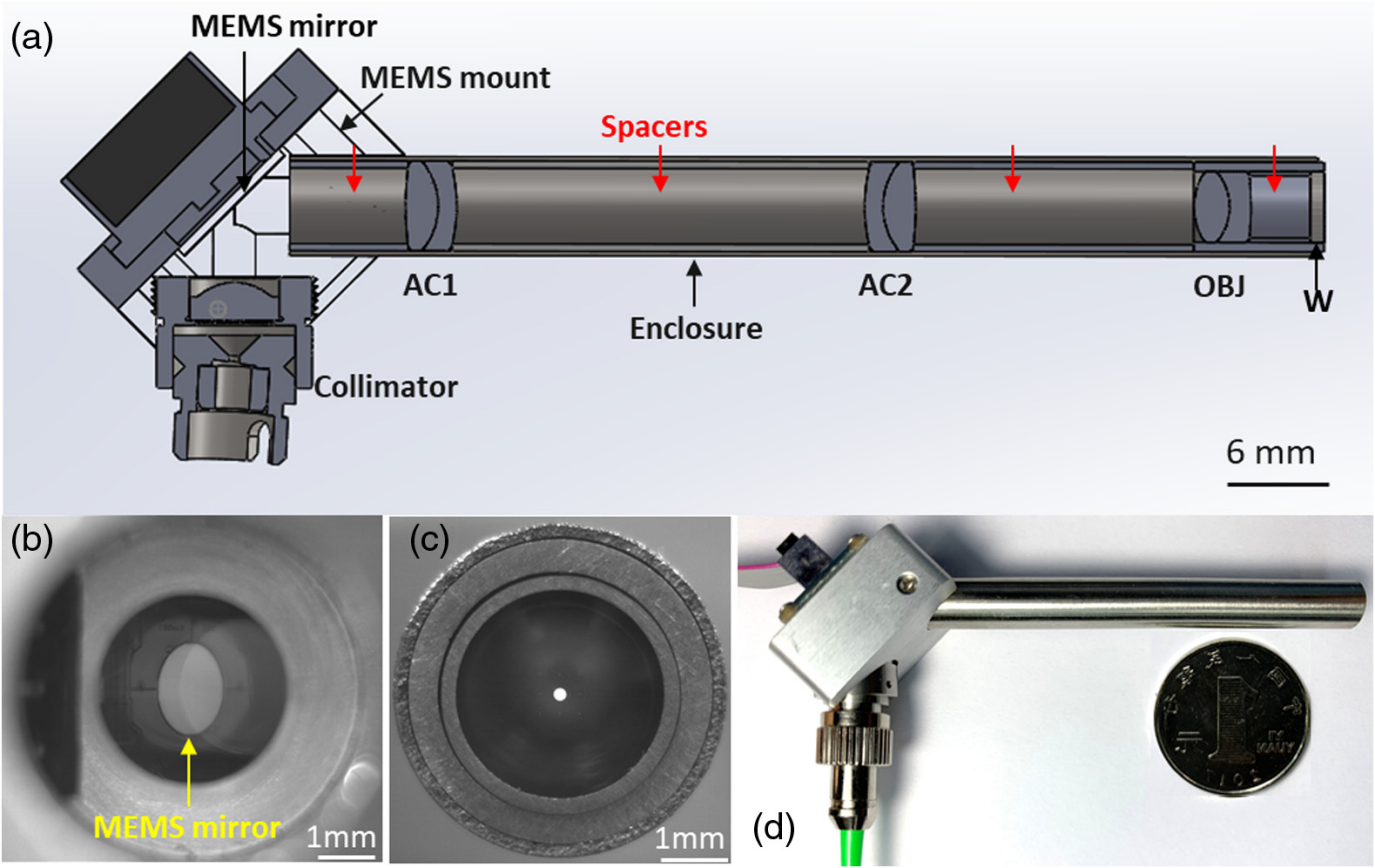
Mechanical design schematic and assembly of the probe. (a) Section view of the optomechanical assembling. AC, achromat; OBJ, objective; W, window. (b) Photograph of the MEMS mirror taken from the hole of the holder reserved for the collimator. (c) Photograph of the tip of the rigid lens tube with the laser on. The focal plane of the camera was tuned to overlap with the enclosure tube’s end face. (d) The photo of the handheld probe assembly.

From the mechanical aspect, the handheld probe is composed of three subassemblies [Fig. 2(a)], i.e., the collimator, the MEMS mirror, and the rigid lens enclosure tube. These subassemblies were integrated through a customized aluminum MEMS mount, which was specially designed and precisely machined to simplify the assembling and alignment, as well as disassembling and replacement of components. The MEMS mirror was fixed on the mount by four screws. Matching threads were tapped in the hole for the collimator, allowing an off-the-shelf aspheric collimator to be installed directly without using set screws or adhesive. The lens enclosure tube is 7-mm diameter and 70-mm long out of the mount. Deployed inside the stainless-steel rigid enclosure tube are three lenses and four pieces of precision-made lens spacers. Spacers were machined precisely according to designed separations between lenses and the MEMS mirror. An antireflection optical window was glued to the distal end of the enclosure tube and sealed to allow convenient disinfection and repeated screening. The working distance was designed to be ∼500  μm from the outer surface of the optical glass window, matching the typical penetration depth of 1310-nm light within oral cavity mucosa. The enclosure tube was fastened to the MEMS mount by two screws; it can be easily replaced by enclosure tubes housing other imaging optics systems adapted to achieve higher magnification, larger FOV, or side-viewing geometry. Figure 2(b) shows a photograph of the MEMS mirror mounted on the 45-deg holder viewed through the collimator opening, revealing the excellent concentricity between the MEMS mirror and the opening (and thus the collimated beam). After complete assembling, the on-axis spot position was checked by setting the MEMS scanner to its initial position. As shown in Fig. 2(c), the output beam spot was centered with respect to the enclosure tube. A photograph of assembled handheld probe is shown in Fig. 2(d), with a Chinese Yuan coin (25-mm diameter) placed aside for comparison. The total cost of the full assembly, including every component displayed in Fig. 2(d) and the MEMS driver (not shown), is ∼$1300, which is much cheaper than conventional galvo-based handheld probes, rotary joint or micromotor-based fiber-optic catheters, or commercial MEMS-based handheld probes (e.g., Thorlabs, OCTH-1300).

### 2.2 System Setup

**Fig. 3:**
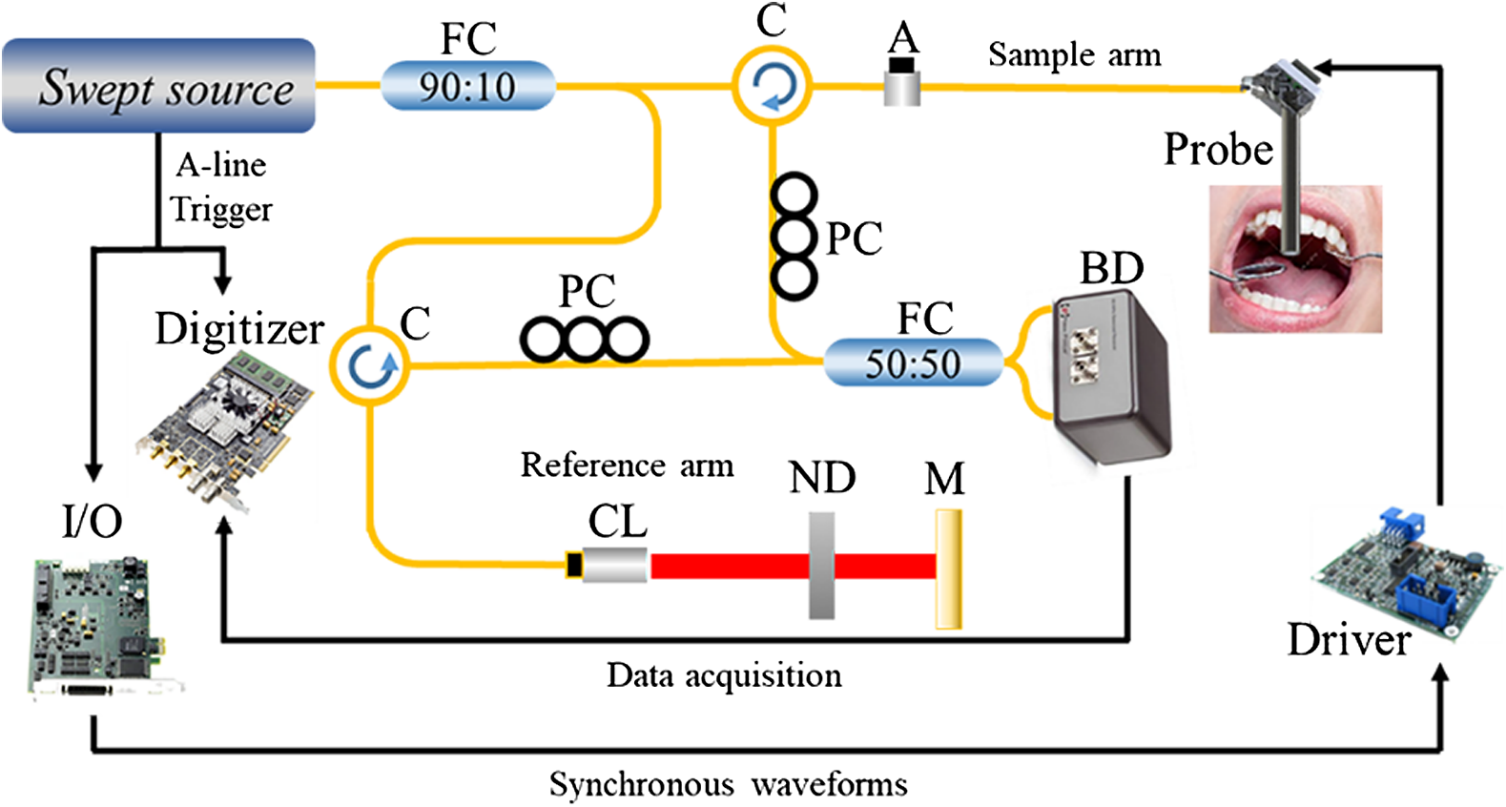
Schematic of the SS-OCT handheld probe system. Light-delivering along optical fibers is indicated by yellow lines, while wires for electronic and control signals are colored black. A, variable fiber optical attenuator; BD, balanced detector; C, circulator; CL, collimator; FC, fiber coupler; M, mirror; ND, neutral density filter; PC, polarization controller.

Details of the home-built benchtop swept source OCT (SS-OCT) system were described in our previous report,^31^ and the system schematic is shown in Fig. 3. Briefly, the swept source (Santec, HSL-20-100-B) used here features a sweep rate of 100 kHz and a central wavelength of 1310 nm with a 3-dB spectral bandwidth of ∼80  nm. Through a coupler of 90:10 split ratio, ∼18  mW out of the output power (∼20  mW in total) is fed into the sample arm and the rest goes into the reference arm. The output power of the probe is controlled by a variable optical attenuator. The MEMS mirror is aluminum coated with a reflectivity of ∼77%, and the maximum output of ∼14  mW is sufficient for *in vivo* imaging. It is electrically driven by a matching driver that amplifies input signals generated by a multifunctional I/O device (PCIe-6321, National Instruments). For B-frame scanning, a single-channel 100-Hz, 9.375-Vpp saw-tooth waveform is generated by the I/O device to drive the fast axis of the MEMS mirror to an angular range of ±4  deg. For 3-D imaging, another channel of similar but slower saw-tooth waveform, typically 0.1 Hz in frequency, is generated by another I/O channel to drive the slow axis. The frequency and amplitude of driving waveforms can be easily tuned to accommodate other scanning speeds and field sizes. Connections between the probe and the OCT system are shown in Fig. 3.

### 2.3 Imaging Procedure and Data Acquisition

All experiments were conducted in accordance with the protocol approved by Ethics Committee of Tianjin Stomatological Hospital in China. Healthy volunteers and volunteers with canker sores and leukoplakia were enrolled for *in vivo* and *ex vivo* imaging studies. Volunteers were informed of experiment contents, and consents were obtained prior to each experiment. For *in vivo* studies, an ergonomic chin support was installed on the optical platform to assist the subjects hold still and, therefore, to minimize involuntary motion artifacts during imaging. The operator held the probe steadily and brought it close until it almost touches the target tissue surface. The distance between the probe tip and the tissue was adjusted according to the real-time OCT images. For *ex vivo* human oral tissues studies, suspected abnormal tissues were harvested from a patient who suffers a white spot on the ventral tongue and then pinned to a wax block for imaging. After data acquisition, the samples were fixed in formalin at once for histopathological processing.

It took about 10 s at a speed of 100 B-frames per second to acquire a 3-D volumetric image consisting of 1024×1000×1000  voxels. To acquire a larger FOV, the operator can slowly and steadily move the probe for a panoramic view. After imaging, the probe was removed and disinfected for reuse according to the endoscope disinfection procedure (submersion in CIDEX OPA solution for 12 min).^32^

## 3 Results

### 3.1 *In vivo* Gingiva and Mucosa Imaging

**Fig. 4:**
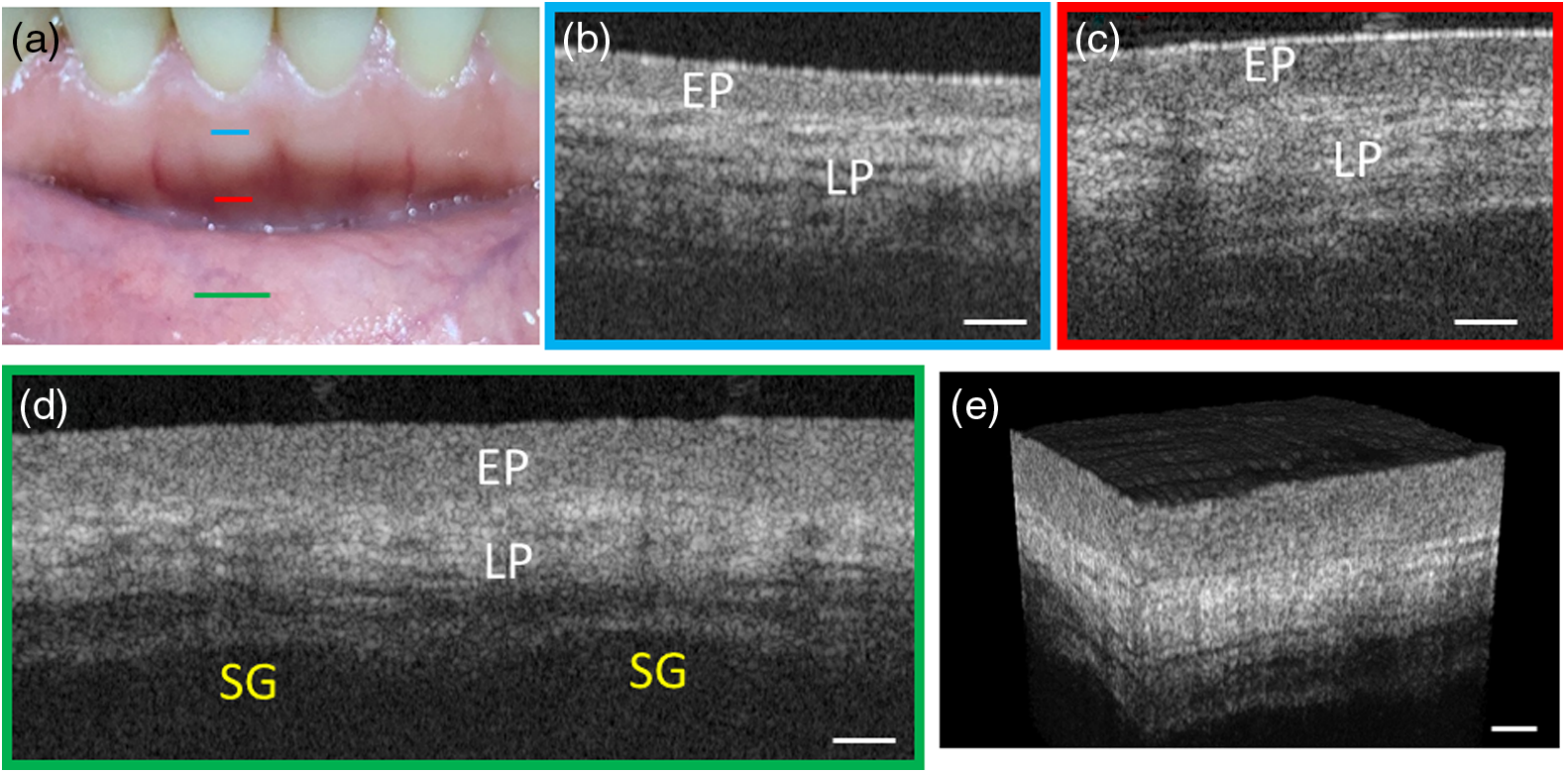
Cross-sectional OCT images of gingiva and oral mucosa. (a) Photograph of gingiva and mucosa that have been imaged. Imaging locations are marked by straight lines, with color corresponding to the border color of (b)–(d). (b) An OCT image of gingiva corresponding to the area. (c) An OCT image of alveolar mucosa. (d) An OCT image of the mucosa of lower inner lip stitched from two single images. (e) 3-D volumetric image of the lower lining mucosa. EP, epithelium propria; LP, lamina propria; SG, salivary glands. Scale bar: 250  μm.

Soft tissues including gingiva and oral mucosa were examined first *in vivo* to demonstrate the probe’s imaging performance. Three color lines in Fig. 4(a) specified the scanning areas and B-frame orientations. Figure 4(b) shows a typical OCT image of the attached gingiva that wrapped the teeth root. The keratinized stratified squamous epithelia propria (EP) and lamina propria (LP) layers were clearly resolved. The LP layer features inhomogeneity and higher backscattering signal due to the abundant dense fibrous tissue. Figure 4(c) shows an OCT image of the alveolar mucosa, which is similar to gingiva except for a thicker EP layer. Moreover, the overall penetration depth in the alveolar mucosa [Fig. 4(c)] was larger than that in gingiva [Fig. 4(b)]. Figure 4(d) displays the OCT image of the lining mucosa of the lower lip. The EP layer is even thicker and the total imaging depth is also deeper than that of gingiva [Fig. 4(b)]. The reason is that alveolar and lining mucosae are covered by nonkeratinized stratified squamous epithelium, whereas attached gingiva is covered by keratinized stratified squamous epithelia, which scatters more strongly and thus is less penetrable for the excitation light.^20^ Saliva glands (SG) are discerned in Fig. 4(d) as two weakly scattering areas with clear upper boundaries (lower boundaries not revealed). Figure 4(e) shows the 3-D volumetric rendering of the lower lining mucosa, which reveals a leather-like microscopic surface texture.

### 3.2 Imaging of Ventral Tongue Leukoplakia

**Fig. 5:**
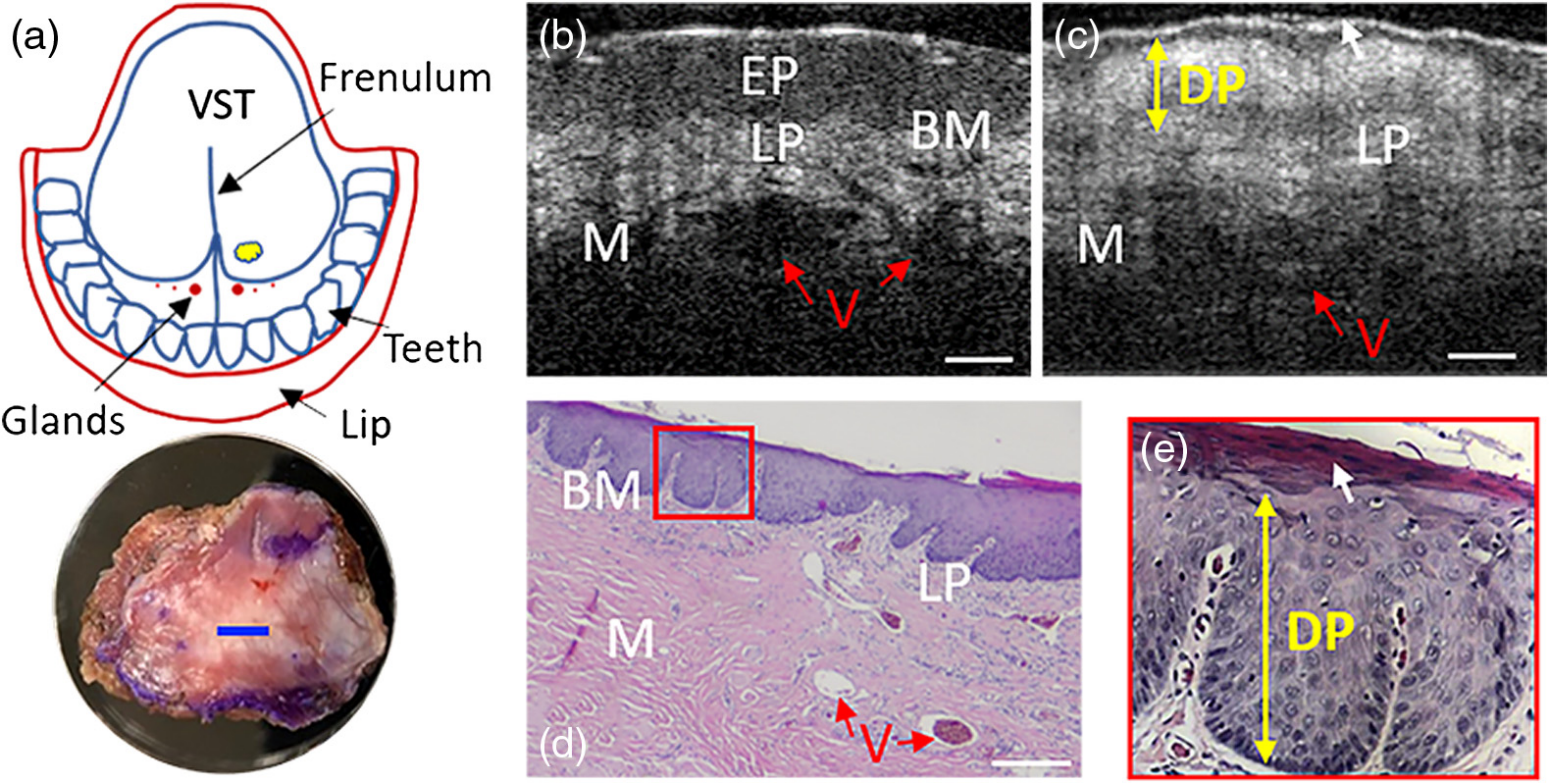
OCT and histological images of normal ventral tongue and ventral tongue leukoplakia. (a) A sketch of the leukoplakia region with the location of tissue resection highlighted by a yellow blob (up) and a close-up photograph of the harvested sample (bottom with the OCT imaging range (2 mm in lateral length) indicated by a blue bar. (b) An OCT image of healthy ventral tongue *in vivo*. (c) An OCT image of resected ventral tongue leukoplakia *ex vivo*. (d) A representative histology micrography of ventral tongue leukoplakia. (e) Close-up of the small region framed by a red rectangle in subfigure (d). BM, basement membrane; DP, dysplasia; EP, epithelium propria; LP, lamina propria; M, muscle; V, blood vessel; VST, ventral surface of tongue. Scale bar: 250  μm.

To demonstrate the probe’s capability of detecting abnormal structures, leukoplakia was studied *ex vivo* for comparison. Leukoplakia refers to a firmly attached white patch on mucosa. Classified as a precancerous lesion with unknown reasons, leukoplakia can only be applied after excluding other possible causes.^33^^,^^34^ A biopsy sample was resected from the leukoplakia area [indicated by a yellow blob in Fig. 5(a)] of the ventral tongue mucosa of a 63-year-old patient and imaged with our OCT probe [with the imaging location marked by a blue line in the close-up photograph of the resected tissue in Fig. 5(a)]. For comparison, ventral tongue mucosa of a healthy volunteer was also imaged at the same location with our probe. The resultant OCT images are shown in Fig. 5(b) (normal) and Fig. 5(c) (leukoplakia), respectively. In Fig. 5(b), main layers including EP, LP, and muscle are clearly discernable even spacing between muscle bundles (ribbon-like weakly scattering pinstripes in the muscle layer), BM, and blood vessel are evident. On the contrary, Fig. 5(c) shows EP with unusual increased intensity and blurred BM. The indicated high scattering layer, dysplasia (DP), suggests the invasion of DP from basal sublayer to at least embryonic sublayer or even mature sublayer (sublayers in EP, from top to bottom are mature layer, embryonic layer, and basal layer, respectively) in EP, which matches well the histology image shown in Fig. 5(d). Figure 5(d) also reveals thick stratum corneum illustrated by white arrows in Figs. 5(b) and 5(d), and normal ventral tongue is generally nonkeratinized as shown in Fig. 5(a). The three images [Figs. 5(b)–5(d)] suggest that DP stems from basal sublayer and grows upward until reaching the mature layer (the most apical sublayer of EP) but do not affect layers below.

### 3.3 *In vivo* Imaging of Canker Sore Recovery

**Fig. 6:**
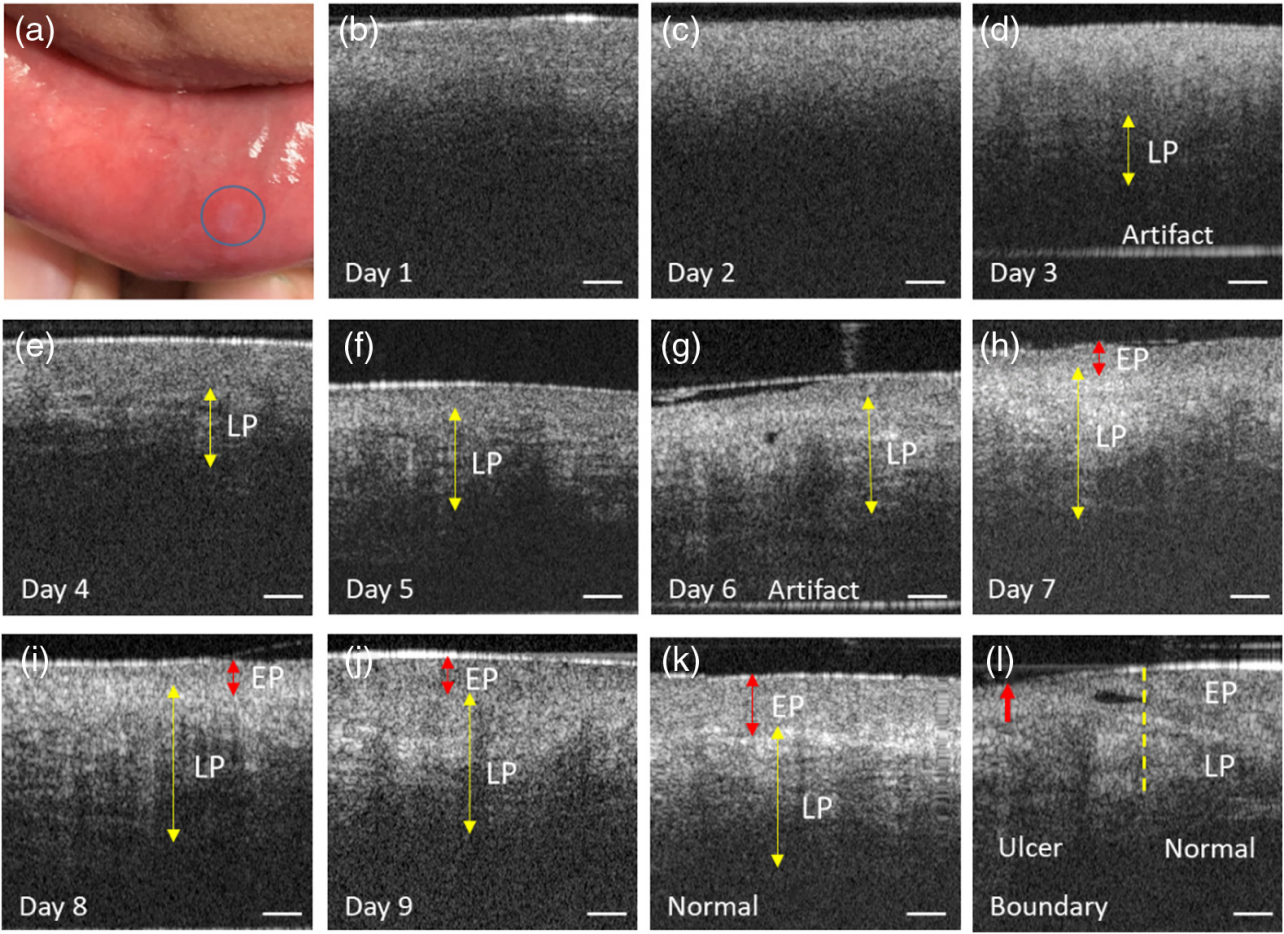
Cross-sectional images of a canker sore throughout the recovery progress. The area marked by the blue circle in (a) is the canker sore. (b)–(j) Intensity images of the canker sores monitored for 9 consecutive days. (k) Normal mucosa of the inner lower lip. (l) Boundary between the canker sores and normal mucosa. The horizontal bright lines in the lowest part of day 3 and day 6 images are artifacts. EP, epithelium propria; LP, lamina propria. Scale bar: 250  μm.

To further validate the OCT probe’s screening capability, the mucosa of a canker sore on the inner lower lip [Fig. 6(a)] of a 24-year-old male volunteer was monitored for 9 consecutive days to track the recovery process, with corresponding representative OCT images juxtaposed in Fig. 6 along with a normal control image for comparison.

While EP and LP layers could be vaguely discerned in the day 1 OCT image [Fig. 6(b)], there are no discernible layered structures in the day 2 image [Fig. 6(c)], which suggests exacerbation of the canker sore on day 2. Starting from day 3, the recovering LP layer starts to emerge in the images, first at deeper locations [∼700  μm deep at day 3, Fig. 6(d)], and then gradually extends into shallower locations through day 4 to day 5 [yellow double arrows, Figs. 6(e) and 6(f)]. On the 6th day, the LP layer has almost entirely recovered, whereas the EP layer is still in the compromised state [Fig. 6(g)]. It takes another 3 days for the EP layer to get restored to the almost normal appearance [Figs. 6(h)–6(j)]. In the day 9 image [Fig. 6(j)], the morphology of both EP and LP layers is very similar to that of normal control [Fig. 6(k)], except that the boundary between EP and LP is not as distinct as in the normal tissue. In the image taken across the canker sore margin [Fig. 6(l)], the lesion area on the left side and the normal tissue on the right side are clearly separable, with the boundary indicated by a yellow dashed line. The superficial surface of the canker sore sagged inward relative to surrounding normal epithelium, as indicated by the retracted upper boundary of the left portion of the OCT image [red arrow, Fig. 6(l)].

### 3.4 *In vivo* Teeth Imaging

**Fig. 7:**
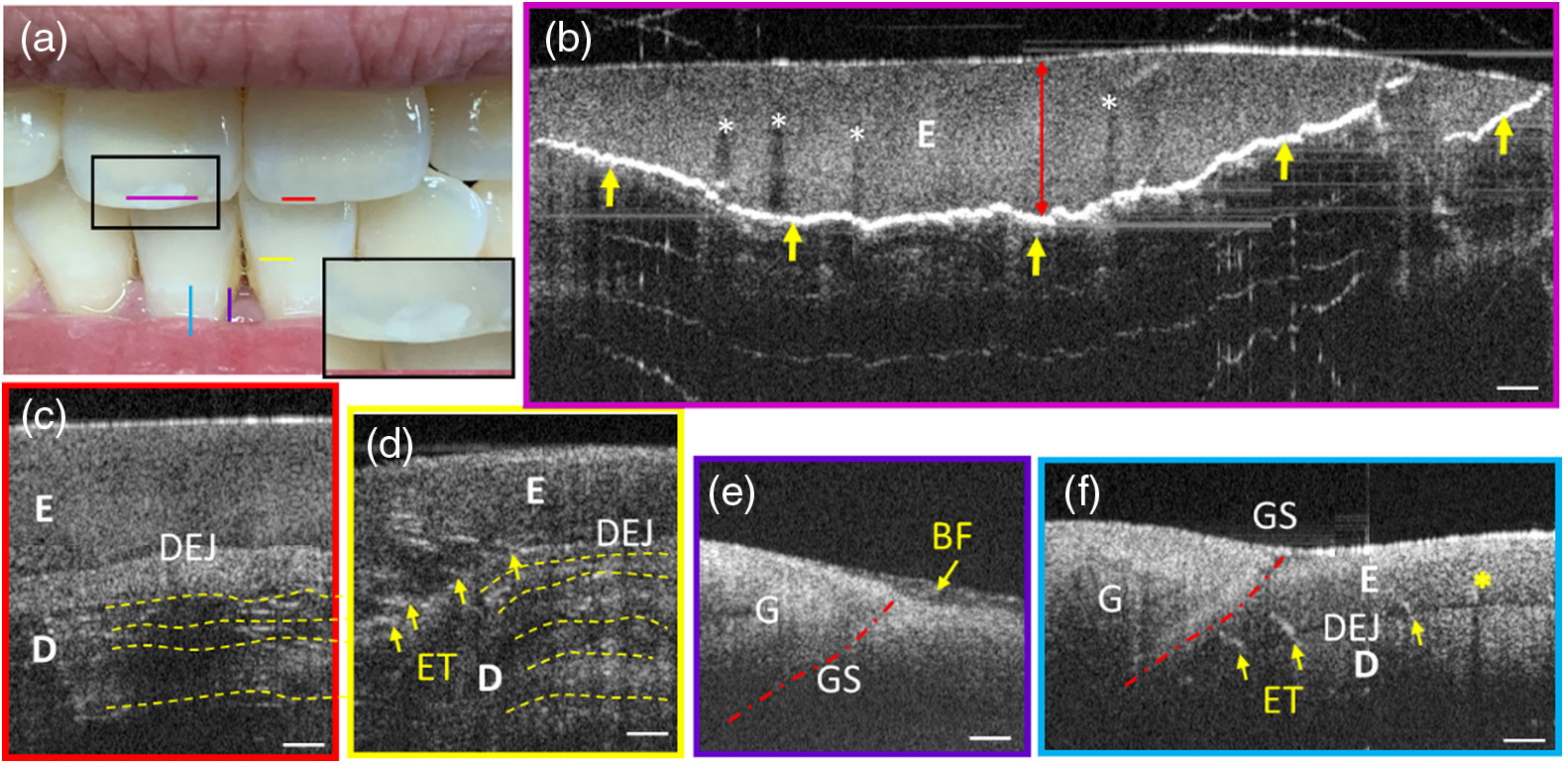
Cross-sectional OCT images of teeth. (a) A photo of teeth under examination. Imaging locations are marked by straight lines with color corresponding to the border color of subfigures (b)–(f). (b) Stitched cross-sectional image of the cracked maxillary central incisor. (c) Cross-sectional image of the healthy right maxillary central incisor. (d) Cross-sectional image of the waist of the mandibular central incisor. (e) Interface between gingiva, a tooth, and biofilm. The red dash dotted curve GS is the gingival sulcus. (f) Junction between gingiva and a tooth (two single images stitched). For all subfigures, BF, biofilm; E, enamel; D, dentin; DEJ, dentin–enamel junction; ET, enamel tuft; G, gingiva; GS, gingival sulcus. Scale bar: 250  μm (Video 1, 8769.198 kb, MP4 [URL: https://doi.org/10.1117/1.JBO.25.4.046003.1]).

Though the handheld probe was originally designed for soft tissues imaging, its versatility had been tested by imaging incisors *in vivo* as an example to examine whether the probe could be used for hard tissues imaging while keeping its high resolution. Five imaging locations were selected on central incisors, marked by colored lines in Fig. 7(a). This volunteer has relatively healthy teeth except for a whitish inner lesion on the left maxillary central incisor [a close-up view shown in the inset of Fig. 7(a)], which resulted from accidently biting a metal chopstick heavily about 3 months ago. To cover the entire lesion, four consecutive FOVs were acquired and stitched together to form a complete cross-sectional OCT image, as shown in Fig. 7(b).

The irregular curve with extremely strong scattering [indicated by yellow arrows, Fig. 7(b)] highlighted the range and the boundary of the hard object-induced crack lesion. The largest thickness [red double arrow, Fig. 7(b)] of this abnormality measures 0.9 mm (assuming a refractive index of ∼1.6 in teeth^35^), which falls below the typical thickness range of the central incisor enamel.^36^^,^^37^ The absence of dentin–enamel junction (DEJ) above the crack boundary also implies that the crack lesion is contained within the enamel layer, as the strong reflection at the crack boundary shadowed the DEJ and dentin layer beneath. In comparison, dentin and DEJ are distinct for the healthy maxillary central incisor [Fig. 7(c)]. Vertically extended low scattering regions within the enamel layer above the crevice were shadows caused by high scattering microcracks^20^ [white asterisks in Fig. 7(b)]. The shadow-free enamel region above the crack boundary shares high similarity in intensity and morphology to the enamel layer of the healthy maxillary central incisor [Fig. 7(c)], suggesting that the enamel above the crack boundary is still normal in general and the whitish visual appearance of the lesion area results mainly from the underlying crevice. In Fig. 7(d), imaging was performed on the waist of the mandibular incisor [yellow line, Fig. 7(a)]. The thickness of the enamel appeared thinner than that close to the cutting edge shown in Fig. 7(c), which matches the known fact that the enamel thickness becomes thinner from the incisal surface to the cervical line (i.e., the neck) of an incisor.^38^ In Figs. 7(c) and 7(d), incremental growth lines (yellow dashed curves) are also discernible in the dentin sublayers. In addition, enamel tufts (ET) are manifest as short ribbon-like structures [yellow arrows, Figs. 7(d) and 7(f)] that scatter sparsely in the DEJ and extend into the enamel.^39^ The junction between a tooth and gingiva was also examined. Biofilm covering the junction can be discerned easily [Fig. 7(e)]. Besides tooth sublayers and features (E, D, DEJ, and ET) described above, also evident in the OCT image are the gingiva sulcus (GS) and a shadow caused by a microcrack [yellow asterisk, Fig. 7(f)].

## 4 Discussions

In this study, a low-cost, ultracompact handheld OCT probe was developed and its great promise for oral maxillofacial tissue imaging was demonstrated. Assuming that the tissue refractive index is ∼1.4, the achieved ∼12  μm axial resolution and ∼9  μm lateral resolution in tissue are well-suited for oral maxillofacial investigation. With the flexibility offered by the long rigid probe tube and the light weight of only 25 g, an operator can easily manipulate and position the probe in the oral cavity as well as on the maxillofacial region. Specifically, to achieve a panoramic extended FOV for clinical convenience, imaging is implemented by manually moving the probe across tissue surface in a similar fashion as handwriting with a pen to produce mosaics for stitching as shown in Figs. 4(d), 7(b), and 7(f). Particularly, raw data of Fig. 7(b) are provided in Video 1 as a demonstration of extended FOV. While the position of tissue surface could vary in the resultant B-frames (due to inevitable relative motion between the tissue and probe surface), such shift between adjacent frames can be easily corrected in postprocessing. Supported by the fast scanning speed of the MEMS mirror, up to 200-Hz B-frame rate (assuming 500 A-lines per frame) can be reached while maintaining a 2-mm-wide scan range with sampling density higher than the Nyquist sampling criterion. Such speed is fast enough to support more panoramic imaging modes with excellent immunity to motion artifacts. For example, another panoramic mode, given 100k-Hz A-line rate of the swept source laser, is to set the B-frame rate to, say 315 Hz (as supported by the MEMS mirror), and the number of A-lines per B-frame to ∼317 so as to cover a ∼1.42  mm (x)×1.42  mm (y)×4 (z)  mm (reduced FOV to meet the Nyquist sampling criterion) volumetric FOV in 1 s. In this way, an operator can move the probe steadily along any direction across the tissue surface, and automated video-mosaicking algorithm specially optimized for such 3-D volumetric OCT data can be developed to generate mosaicked *en face* image over an extended FOV to help locate and delineate subepithelial lesions or abnormalities.^40^^,^^41^ Panoramic modes with other FOV and sampling density combinations can be tailored to fit specific applications representing a critical future direction for clinical applications.

In most previous MEMS-based designs,^24^^–^^26^^,^^29^^,^^30^ the MEMS scanner was installed at the distal end of the probe, and thus the ∼100  V high-driving voltage might potentially cause electric hazard and safety issues. The proposed design instead moves the MEMS scanner to the proximal end, farther away from the tissue, which alleviates the safety concern and also simplifies the lens tube design, making the lens tube readily switchable for other optics settings (e.g., side-viewing mode and/or different working distances).

The performance and versatility of the probe herein reported are demonstrated by real-time, volumetric imaging of various oral maxillofacial tissues. Microscopic structural features of gingival tissues, oral lining mucosa, and teeth, both healthy and compromised, are distinctly resolved with our OCT probe. For example, the hidden crack on a tooth is hardly distinguishable from demineralization based on visual inspection since both appear as whitish spots on the tooth surface.^20^ However, these two defects look totally different in OCT images. While demineralization is manifested as strong scattering throughout the affected enamel,^20^ the hard-object-induced inner crack exhibits normal enamel signal with high scattering signal localized to near the crack boundary [Fig. 7(b)]. For the canker sore experiment, although histology correlation is absent, the consecutive observations reveal the recovery process, and the differences day-by-day are clearly discerned, demonstrating the strong potential of the OCT probe for routine screening and biopsy guidance.

In addition to imaging performance, price is another important practical concern when designing an OCT probe for clinic translation. In this report, the customized probe costs $1300, and the price can be further reduced in mass production. A commercial MEMS-based OCT probe with much larger size (157.9-mm long and 31.3-mm diameter) and lower imaging speed (28  frame/s) is priced > $10k on Thorlabs.

The current design can be easily adapted into side-viewing geometry by deploying at the distal end at 45-deg (or other angles) mirror, which reflects the beam sideways. Further, the reflection mirror can be rotated with a high-speed miniature DC motor to scan the beam in a helical fashion to form an extended FOV.^42^^,^^43^ The 500-μm working distance of the current design is optimized for mucosa penetration, and the penetration depth in teeth can reach as deep as ∼2  mm. Dynamic focus tuning through a solid tunable lens^44^^–^^46^ or shape memory alloy^47^ can also be implemented in future designs to better accommodate different imaging setups.
